# Case Report: Acute Renal Infarction in a Child With Coarctation of Aorta

**DOI:** 10.3389/fped.2021.707560

**Published:** 2021-08-05

**Authors:** Qing-Yun Zhang, Min-Hua Tseng, Jhao-Jhuang Ding, Jing-Long Huang

**Affiliations:** ^1^Department of Pediatrics, Xiamen Chang Gung Hospital, Xiamen, China; ^2^Division of Nephrology, Department of Pediatrics, Chang Gung Memorial Hospital and Chang Gung University, Taoyuan, Taiwan; ^3^Department of Pediatrics, National Defense Medical Center, Tri-Service General Hospital, Taipei, Taiwan; ^4^Division of Allergy, Asthma, and Rheumatology, Department of Pediatrics, Chang Gung Memorial Hospital and Chang Gung University, Taoyuan, Taiwan

**Keywords:** renal infarction, coarcted aneurysm, pediatrics, thrombus, glomerulonephritis

## Abstract

Renal arterial infarction can present with hematuria, proteinuria, and hypertension, features often linked to glomerular disease. An aortic aneurysm is an extraordinarily rare complication of coarctation of the aorta. Acute renal infarction caused by emboli from the aortic aneurysm is a possible complication that has not been reported. We herein report a 10-year-old boy who presented with hematuria, proteinuria, hypertension, and skin rashes on both lower extremities mimicking acute glomerulonephritis but actually resulting from acute renal infarction caused by a coarcted aneurysm-associated thrombus. He was successfully treated with surgical excision of the coarcted aorta and aneurysm followed by subcutaneous low molecular weight heparin without recurrence.

## Introduction

Renal infarction is rare in the pediatric population (1–3) and has the potential for significant morbidity if recognition is delayed. Differential diagnoses include glomerulonephritis, pyelonephritis, gastroenteritis, or renal stones (4). Although cardioembolic disease, fibromuscular dysplasia, renal artery thrombosis, and hypercoagulable states are the most common causes of acute renal infarction, other etiologies that can predispose to emboli formation such as aortic aneurysm should be considered.

Herein, we report a case of acute renal infarction caused by an aneurysm-associated thrombus in a child with untreated aortic coarctation. He was managed successfully with surgical intervention and low molecular weight heparin.

## Case Description

A previously healthy 10-year-old boy presented with a 1-day history of fever, abdominal pain, and skin rashes on the lower extremities. The abdominal pain was described as burning and persistent on the left upper abdomen. There was no vomiting, diarrhea, trauma, dysuria, or abnormal urinary frequency. The family history was unremarkable. His blood pressure was 146/92 mmHg, heart rate 150 beats/min, respiratory rate 36 breaths/min, and body temperature 38.0°C. Physical examination revealed mild left upper quadrant abdominal tenderness with equivocal knocking pain on the left costovertebral region and non-blanchable rashes on bilateral lower extremities.

**Figure 1:**
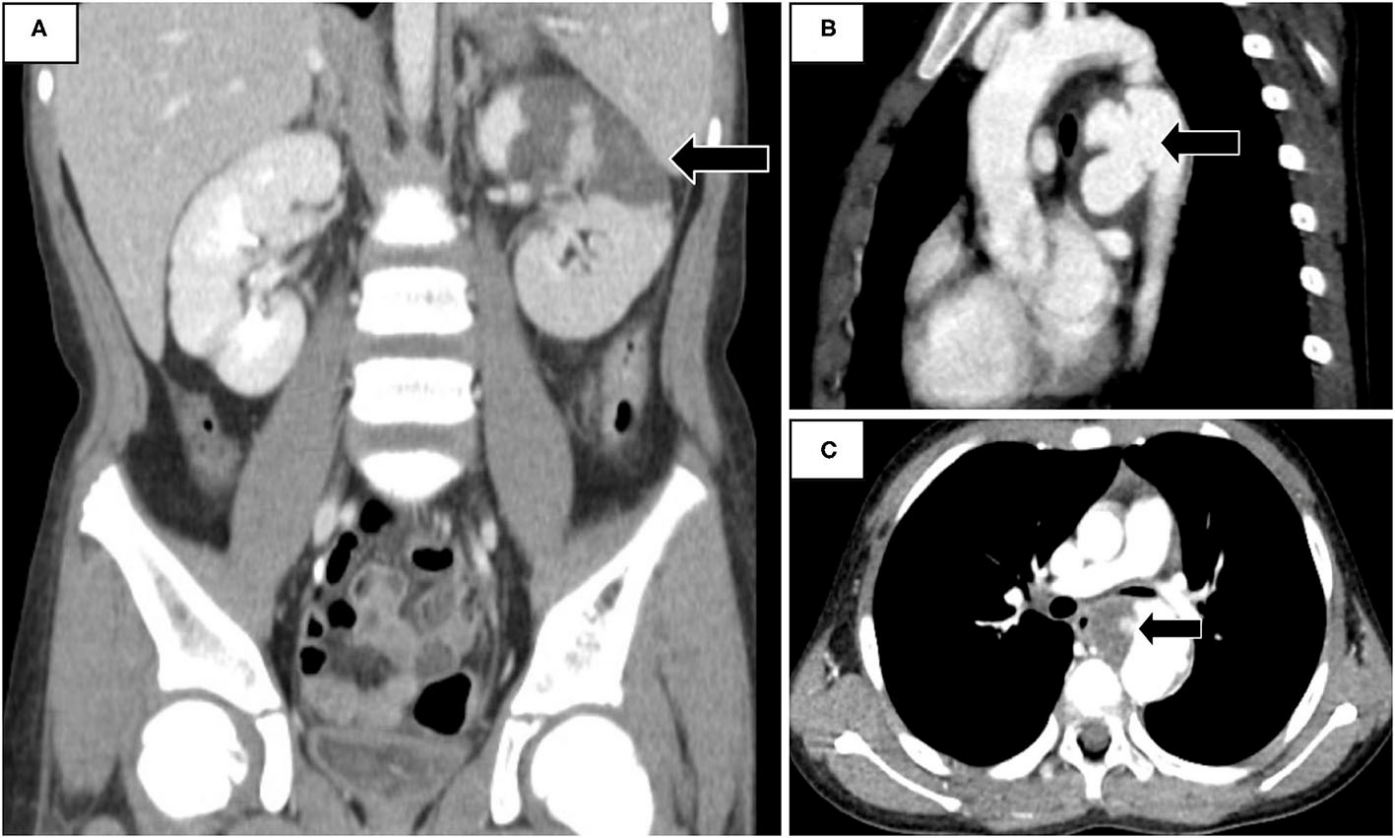
**(A)** Abdominal Computed tomography (CT) revealed infarction in left kidney. **(B)** CT angiogram demonstrated a coarcted aneurysm at the descending aorta. **(C)** Complicated intra-aneurysmal mural thrombus formation with ulceration.

The laboratory studies showed leukocytosis, anemia, upper limit creatinine, elevated C-reactive protein, and lactate dehydrogenase. Urine analysis demonstrated the presence of microscopic hematuria and proteinuria, with pyuria (Table 1). His fever did not respond to empiric antibiotics, and both urine and blood cultures were negative. Abdominal computed tomography (CT) demonstrated renal infarction by the presence of wedge-shaped perfusion defects in the left kidney (Figure 1A). Hypercoagulatory, rheumatologic diseases investigations and an electrocardiogram did not reveal abnormalities. Echocardiogram and CT angiogram revealed coarctation of the descending aorta (Figure 1B) and a 35-mm aneurysm with an intra-aneurysmal mural thrombus distal to the coarctation (Figure 1C). His fever and rashes subsided after an uneventful surgical excision of the coarctation and aneurysm. The intraoperative specimen culture was negative. After surgery, his laboratory data, including white blood cells, C-reactive protein, lactate dehydrogenase, and abnormal urinary sediments returned to normal (Table 1). His renal infarction was treated with subcutaneous low molecular weight heparin followed by 6 months of oral anticoagulant therapy. At 5-year follow-up, he remained normotensive without recurrence.

**Table 1:** Laboratory data.

	**At presentation September 2013**	**Follow-up December 2013**
White blood cells (/μl)	24,800	12,000
Neutrophil (%)	89.5	65.5
Lymphocyte (%)	5.5	19.5
Monocyte (%)	3.5	3.5
Eosinophil (%)	0	0
Hb (g/dl)	10	12
Platelet (×10^3^/μl)	214	160
Blood urea nitrogen (mg/dl)	9.8	12
Creatinine (mg/dl)	0.69	0.60
C-reactive protein (mg/dl)	10.55	0.5
Lactate dehydrogenase (U/L)	1,200	80
Urine analysis		
Specific gravity	1.030	1.008
Protein	2+	–
Glucose	1+	–
Ketone	1+	–
Occult blood	3+	–
Red blood cell (NR: <20/μl)	>500	10
White blood cell (NR: <30/μl)	80	15
Epithelial cell (/μl)	10	5
Blood culture	No growth	NA
Urine culture	No growth	NA
Tissue culture of the aneurysm	No growth	NA

*Hb, hemoglobin; NR, normal range*.

## Discussion

To the best of our knowledge, our finding of a coarcted aneurysm-associated thrombus causing acute renal infarction has not been reported before. This 10-year-old boy presented with fever, rashes, and abdominal pain resulting from renal infarction caused by a coarcted aneurysm. The characteristics of hematuria, proteinuria, hypertension, and rashes on extremities mimic the features of Henoch–Schönlein purpura. Nevertheless, the left costovertebral knocking pain, fever, and elevated serum lactate dehydrogenase and C-reactive protein indicated the possibility of renal infarction (3, 5, 6). An abdominal CT confirmed the diagnosis. Besides thrombolytic or anticoagulant treatment, investigating the underlying etiology should be the focus in order to prevent recurrence of renal infarction. The most common causes of renal infarction are cardiogenic, including arrhythmia, cardiomyopathy, valvular heart diseases, and thrombi from the suprarenal aorta or left ventricle followed by renal artery injury and hypercoagulable state (6, 7). Diagnostic testings including intra-cardiac diseases, hypercoagulable conditions, rheumatologic diseases, and images for the vascular abnormalities are essential for uncovering the underlying etiology. Computed tomographic angiogram demonstrated a descending aortic coarctation-associated aneurysm and complicated intra-aneurysmal mural thrombus formation with ulceration.

Coarctation of the aorta (CoA) is one of the common congenital heart diseases, but only a few patients develop late complications, including hypertension, cardiac failure, coronary artery disease, and aneurysm (8). An aortic aneurysm is a lethal but potentially easily misdiagnosed sequela of corrected aortic coarctation. The prevalence of coarcted aneurysms is not rare, around 5.8%, but almost rises after surgical repair (9). Renal manifestation as the initial presentation of aortic aneurysm is very uncommon and carries diagnostic challenges in patients with previously unrecognized CoA (10). To our knowledge, coarctation-associated aneurysms have been reported in only two pediatric patients who had no prior repair (11, 12). The exact pathogenesis of aneurysms formation of untreated CoA remains unclear. Two hypotheses have been proposed to explain the development of a coarcted aneurysm: first, weaknesses of the aortic wall, caused by friable ductus tissue and coarctation-associated pressure gradient (8) and second, a mycotic aneurysm, also resulting from post stenotic pressure gradient, causing infectious destruction of the vascular wall.

In conclusion, our case highlights that acute renal infarction could present with features mimicking the characteristics of acute glomerulonephritis and intra-aortic thrombus caused by coarcted aorta should be considered as a possible origin of the renal emboli. Early diagnosis of the coarcted aneurysm with timely surgical intervention is essential to avoid catastrophic complications.

## Data Availability Statement

The original contributions presented in the study are included in the article/supplementary material, further inquiries can be directed to the corresponding author.

## Ethics Statement

The studies involving human participants were reviewed and approved by the Ethics Committee on Human Studies at Chang Gung Memorial Hospital. Written informed consent to participate in this study was provided by the participants' legal guardian/next of kin.

## Author Contributions

M-HT and J-JD contributed to patient's care. Q-YZ, M-HT, and J-JD wrote the first draft of the manuscript. J-LH contributed to the final version of the manuscript. All the authors contributed to manuscript revision, read, and approved the submitted version.

## Conflict of Interest

The authors declare that the research was conducted in the absence of any commercial or financial relationships that could be construed as a potential conflict of interest.

## Publisher's Note

All claims expressed in this article are solely those of the authors and do not necessarily represent those of their affiliated organizations, or those of the publisher, the editors and the reviewers. Any product that may be evaluated in this article, or claim that may be made by its manufacturer, is not guaranteed or endorsed by the publisher.
